# Conducting clinical research in the era of the COVID-19 pandemic: Challenges and lessons for Speech-Language Pathology and Audiology research

**DOI:** 10.4102/sajcd.v69i2.898

**Published:** 2022-07-18

**Authors:** Katijah Khoza-Shangase, Nomfundo Monroe, Ben Sebothoma

**Affiliations:** 1 Department of Audiology, Faculty of Humanities, University of the Witwatersrand, Johannesburg, South Africa

**Keywords:** audiology, challenges, clinical research, COVID-19, ethical considerations, lessons, Speech-Language Pathology, South Africa

## Abstract

**Background:**

The novel coronavirus disease 2019 (COVID-19) presented new and unanticipated challenges to the academic training and performance of clinical research at undergraduate and postgraduate levels of training. This highlighted the need for reimagining research designs and methods to ensure continued generation of knowledge – a core function of a research-intensive university. Whilst adhering to government regulations geared towards protecting both the research participants and researchers, innovative research methods are required.

**Objective:**

The purpose of this scoping review is to explore published evidence on innovative clinical research methods and processes employed during COVID-19 and to document challenges encountered and lessons that the fields of Speech-Language Pathology and Audiology can learn.

**Methods:**

Electronic bibliographic databases including Science Direct, PubMed, Scopus, MEDLINE, ProQuest were searched to identify peer-reviewed publications, published in English, between 2019 and 2021, related to innovative clinical research methods and processes applied where in-person contact is regulated.

**Results:**

Significant challenges with conducting research in the COVID-19 era were identified, with important lessons learned and numerous opportunities that have relevance for this pandemic era and beyond. These findings are presented under 10 themes that emerged that highlight important considerations for research methods and processes during a pandemic and beyond. The findings of this study also raise implications for telehealth from which low- and middle-income countries (LMICs), where resource challenges exist, can benefit.

**Conclusion:**

Challenges and opportunities identified in this review have relevance for the field of Speech-Language Pathology and Audiology as far as current and future (beyond COVID-19) clinical research planning is concerned.

## Introduction

Coronavirus disease 2019 (COVID-19), originally recorded in Wuhan, China in 2019, has become a global pandemic that has significantly impacted how the world lives and functions (Perez, Perez, & Roman, 2020), as declared by the World Health Organization (WHO) on the 11th March 2020. Within academia, the pandemic has impacted how teaching and learning and research activities are conducted, with challenges caused by the measures put in place to curb the spread of the virus influencing all decisions made (Khoza-Shangase, Moroe, & Neille, 2021; Perez et al., 2020; Sebothoma, Khoza-Shangase, Masege, & Mol, 2021). As far as research is concerned, significant challenges have been encountered in terms of the planning of clinical research designs, sourcing of funding for research as research priorities change, as well as significant questions around ethical clearance of studies conducted during the COVID-19 pandemic. Questions around ethical clearance have had ethical committees and institutional research review boards challenged by their mandate to facilitate ethical research whilst adhering to health and safety regulations implemented to protect and safeguard the safety of research participants and researchers (Beach et al., 2020; Lumeng et al., 2020; Perez et al., 2020; Sebothoma et al., 2021; Wieten, Burgart, & Cho, 2020). Weiner, Balasubramaniam, Shah and Javier (2020) believed that the impact that the COVID-19 pandemic had on general research and on research specific to the pandemic raises numerous important factors: (1) the significance of research, (2) challenges of research, especially during public health emergencies (PHEs) and (3) resources and opportunities towards improving research to become more efficient and cost effective. Furthermore, Bailey, Black and Swanton (2020) argued that the COVID-19 pandemic provided an opportunity and renewed momentum for innovative approaches to research within a restrictive environment.

Evidence indicates that the pandemic has become a significant threat in low- and middle-income countries (LMICs), with the African continent, not being spared from the negative impact (Lone & Ahmad, 2020). Although Africa was the last continent to be affected by COVID-19, the World Economic Forum (2020) predicted that, as the most vulnerable continent, COVID-19 will have major impacts in Africa. As of 26/01/2022, the WHO reports that the 47 African countries are affected, with 10 633 981 cumulative cases and 236 399 deaths because of COVID-19 – with South Africa contributing the most to these numbers, with 3 585 888 confirmed cases and 94 625 deaths (WHO, 2022). With the new COVID-19 variants, including the current omicron, that are continuously creating challenges for the healthcare, educational and economic sectors, innovative and proactive models of survival and productivity are required. This article focuses on conducting clinical research, which is one of the areas requiring deliberation. The large number of immunocompromised populations within a poor healthcare system, in the presence of poor social determinants of health and high burdens of diseases, place the African continent at a greater risk of severe COVID-19 pandemic impact (Lone & Ahmad, 2020; The World Economic Forum, 2020). The World Economic Forum (2020) argued that these conditions could make controlling the pandemic and managing its consequences significantly challenging. With the general lack of access to vaccines coupled with vaccine hesitancy, as well as the absence of a treatment drug currently available for COVID-19, application of non-pharmaceutical measures to contain the spread of the virus remains the only measure available. These measures include national lockdowns and travel restrictions, hand washing and sanitisation, social distancing, isolation and quarantine, as well as community containment. These measures – have a serious impact on teaching, learning, as well as the performance of research (Khoza-Shangase et al., 2021, Maluleke & Khoza-Shangase submitted).

Soon after the national lockdowns were instituted, higher education programmes and their ethical committees and institutional research review boards had to make decisions about what and how research should be conducted as part of the required knowledge generation of higher education institutions. This was also done as part of collating evidence around the COVID-19 pandemic as a disease. Perez et al. (2020) highlighted that, as far as research is concerned, COVID-19 has also inspired new studies that are aimed at learning about the virus and its effects, described and defined the affected patient populations and established the efficacy of available interventions (vaccines and treatment drugs). This necessitated a rapid response to newly submitted research proposals, where participant–researcher interactions had to be considered and where general research approaches had to be revised to suit the new normal conditions with the pandemic. In the United States, Perez et al. (2020) reported that unexpected divergences to standard protocols became unescapable because of the inability of participants to attend research sites for data collection following lockdowns, travel restrictions, quarantine requirements, etc.. Furthermore, some research studies were postponed to adhere to social distancing and to minimise costs linked to personal protection equipment (PPE), with research fellows and general research staff ordered to ‘work from home’ (remote work). The travel restrictions also impacted the access to research tools that are most often imported, particularly in LMICs. Lumeng et al. (2020) further reported on how COVID-19 has had a significant effect on the academic research enterprise in the United States, with numerous research institutions shutting down their research laboratories, adjourning fieldwork and stopping a number of human research initiatives. This led to the cessation of over 80% of all on-site research activity – with research in the basic and natural sciences gradually resuming activities over the course of a few months (Lumeng et al., 2020). The research activities that resumed are those that were deemed to present relatively low risk for virus transmission, under strict adherence to non-pharmaceutical COVID-19 intervention measures (i.e. social distancing).

Within the South African context, the Speech-Language Pathology and Audiology professions, as healthcare professionals, were right in the middle of the previously outlined challenges as well, and thus innovative approaches were required to continue to conduct research under uncertain times, with an indefinite time period. Thus, emergency clinical research plans and policies had to be formulated, alongside emergency online teaching methods. Research plans that still allowed for research questions to be answered whilst adhering to ethical principles such as informed consent, data collection visits, assessments and evaluation procedures, health and safety monitoring, research design monitoring, etc., – whilst protecting researchers and participants in accordance with published and promulgated regulations of the country had to be implemented. As this was a novel situation with challenges and potential opportunities for learning for future innovative clinical research practice, the current scoping review aimed to explore published evidence on innovative clinical research methods employed during COVID-19 to document challenges encountered and lessons that can be learned in the field of Speech-Language Pathology and Audiology. This study is also important because COVID-19 may last longer and perhaps precede other pandemics (Jayaweera et al., 2021).

## Methodology

Levac, Colquhoun and O’Brien’s (2010) scoping review methodology was adopted for this study, with the research team consisting of three researchers working as researchers and research supervisors in Speech-Language Pathology and Audiology university training programmes in South Africa. The researchers came to an agreement on a broad research question that was the focus of the scoping review and on the global study protocol, including specification of Medical Subject Headings (MeSH) terms, keywords, phrases and selection of databases to be searched. For this scoping review, the Arksey and O’Malley’s (2005) framework was adopted, thus following the five key phases of (1) identifying the research question, (2) isolating relevant publications, (3) study selection, (4) charting the data and (5) collating, summarising and reporting the results.

### Research question

The broad question that guided the current scoping review was, ‘what has been published about conducting clinical research during the COVID-19 pandemic?’ This line of enquiry was directed by the challenges presented by COVID-19 in conducting and supervising research in Speech-Language Pathology and Audiology during a time where non-pharmaceutical COVID-19 interventions negatively impacted standard practice and where innovative methods had to be explored and re-imagining future (post-COVID) research practice was called for. The researchers aimed to perform this review to document both challenges and opportunities presented by COVID-19 to research practice, in a context that is resource constrained where opportunities might expand access to participants and indigenous knowledge that might currently be inaccessible within the South African Speech-Language Pathology and Audiology professions. In addition, guided by Daudt, Van Mossel and Scott’s (2013) definition of the value of scoping reviews, the current scoping review also discovered the kinds and sources of evidence obtainable on the stated research question with findings raising implications for conducting clinical research during the COVID-19 pandemic, involving all stages of research including research reviews, ethical clearance and so on.

### Data sources and search strategy

The initial search was carried out in December 2021 in the following five electronic databases: Medline, ProQuest, PubMed, Science Direct and Scopus. These five databases were chosen as they are deemed to be comprehensive and cover publications considering conducting clinical research during COVID-19 by healthcare practitioners. The selected studies were restricted to those published in English from the year of the COVID-19 advent, 2019 onwards, with a focus on clinical research. The search consisted of the following terms: conducting research, clinical research, COVID-19, challenges, opportunities and lessons.

### Resources

A total of 15 citations, as depicted in Table 1, were finally included in the analysis. An additional final search of the five listed bibliographic databases was performed in January 2022 to make sure that any new publications post the initial search were also identified. No new hits were identified.

**TABLE 1:** Summary of studies included in the scoping review documenting clinical research challenges and opportunities during COVID-19.

Authors and date	Title	Challenges(s)	Opportunities or recommendations
(Bailey et al., 2020)	Cancer Research: The Lessons to Learn from COVID-19	*Participant recruitment challenges*	Incorporation of remote working practices, for example, adoption of telemedicine, community visits
		Fixed sites – distance, time commitments and incentives, sampling bias	Decentralisation of trial centres to remote sites – will improve accessibility to patients and reduce the need to travel.
		*Remote working practices to reduce inefficiency required*	Mailing IPs to patients’ homes and supervising IP administration using videoconferencing technology.
		Difficulty in ensuring proper administration of oral investigational products (IP) without hospital visits: patients may frequently miss doses or take two doses of the IPs at once.	
		*Using technology to galvanise recruitment needed*	Enhanced electronic institutional review board (IRB) communications, the standard practice of e-signatures and remote training considered
		*Flexibility of protocol deviations and trial design required*	Work during the pandemic has been disseminated quickly, with many researchers using preprint servers to publish their work.
		*Trial Approval process requires relooking*	Maintaining standards key in this high-speed publishing, for example, transparency in data sources and analytic methods (including code), reproducibility and robust peer review must still occur.
(Fleming, Labriola, & Wittes, 2020)	Conducting Clinical Research During the COVID-19 Pandemic: Protecting Scientific integrity	Risk of bias from nonadherence	Healthcare workers make home visits whilst wearing personal protective equipment
		Potential delay or pause in enrolment of participants	Later re-initiation of enrolment to achieve protocol-specified statistical power can begin after the study team judges that it can adequately manage risks of COVID-19.
		Interruption of delivery of the intervention and study assessments at a site	Maintain contact with participants for retention after the intensity of the outbreak has decreased.
		Incomplete data	Maintain a list of patients whose participation has been adversely affected, along with the consequences.
		Disruption to data collection procedures	All changes to data collection should be well documented.
		Revision of the statistical methods planned	Flexibility may be necessary in terms of intervals of calendar time, termination of research at near completion
			Changes should be reviewed by appropriate committee
		Analytical issues in protecting trial integrity	Valid statistical approaches should guide the presentation of results If data are collected during the period of severe disruption in a manner different from the approach originally planned, the analysis could stratify the data by the method of collection. Along with prespecified primary analyses, sensitivity analyses, prespecified and post hoc, should be presented to assess the robustness of results. Analyses should address the influence of missing data and of deviations from protocol-specified levels of adherence (because of COVID-19).
(Shamsuddin, Sheikh, & Keers, 2021)	Conducting Research Using Online Workshops During COVID-19: Lessons for and Beyond the Pandemic	Practical and methodological challenges in running workshops online	*Sampling bias solution* Provide internet routers to participants in low- and middle-income countries (LMICs)
		Sampling may be biased to those with internet access, particularly in low- and middle-income countries	*Addressing online recording* Adhere to the principles of respect for persons, beneficence and justice. Adhere to precautions for data collection, where participant privacy and confidentiality are of paramount importance State clearly to the IRB the intention to record online and select a suitable recording tool
		Guidance on the ethical implications for recording online (recording of sessions, informed consent)	*Ensuring informed consent* Make it clear in obtaining consent that recordings cannot be removed after participation, limiting the possibility of varied consent for recording. A clear confidentiality statement must be included in participant information sheets and consent forms. Such information sheets should also prohibit recording the workshop session using participants’ own devices.
		Storage of recordings	*Storage and destruction of recordings* Consider whether to use software external to the web conferencing system or alternatively the web conferencing system’s built-in function. Be well versed in the recording facility’s privacy policy as recordings are often stored on the host provider’s platform (i.e. cloud storage) Use password protection to enhance data security Data destruction must be ensured
		Rapport amongst participants as well as between participants and the researcher	*Rapport* Apply proactive strategies in supporting the success of an online workshop, which additionally builds researcher–participant rapport.
(Weiner et al., 2020)	The COVID-19 impact on research, lessons learned from COVID-19 research, implications for paediatric research	Challenges linked to novel approaches and high-quality research	*Novel approaches and high-quality research:* Have appropriate study designs, collaboration, patient registries, automated data collection, artificial intelligence, data sharing and ongoing consideration of appropriate regulatory approval processes
		Time-efficiency challenges	*Time efficiency* During public health emergencies (PHE) or disasters, crisis standards for research should be considered along with ongoing and just-in-time PHE or disaster training for researchers willing to share information that could be leveraged at the time of crisis.
		Cost-effective research	*Cost-effective research* A dedicated funded core workforce of PHE or disaster multidisciplinary researchers and funded infrastructure should be considered, to strategise, consult, review, monitor, interpret studies, guide appropriate clinical use of data and inform decisions regarding effective use of resources for PHE or disaster research.
(Park et al., 2021)	How COVID-19 has fundamentally changed clinical research in global health	Quality of research during COVID-19	A balance must be struck between quickly disseminating data via preprint servers and ensuring that the work is scientifically credible.
(Cagnazzo et al., 2021)	Lessons learned from COVID-19 for clinical research operations in Italy: what have we learned and what can we apply in the future?	Study activation challenges	Simplified approval methods recommended for COVID-19 trials can be maintained beyond the emergency period and applied to different types of clinical research (interventional trials for drugs or medical devices, observational or epidemiologic studies) Use of electronic submission for applications for authorisation and of electronic or digital signature for contracts with sites recommended
		Patient participation challenges	Consider the following alternative measures to enhance patient participation in clinical trials: Facilitate remote patient visits (e.g. video, telemedicine, phone) Incentivise the possibility to perform procedures at the patient’s home – home visits (e.g. blood sample taking, drug administration, questionnaires) whilst ensuring the patient’s anonymity
		Study monitoring challenges	Extend reimbursement of expenses (travel, examinations, procedures) to patients and caregivers without limitation to rare disease clinical trials only
		Research support professionals’ challenges	Facilitate remote monitoring of the study and source data verification Facilitate the implementation of validated electronic medical records and make them available remotely to authorised personnel
		Data protection challenges	Take measures to facilitate the inclusion of adequately prepared and remunerated professionals dedicated to the management of the clinical trial and the collection of the data (e.g. data manager or study coordinator) in the site organogram Explore possibilities of remote informed consent administration
		Research funding and appropriate infrastructure	Funding originating from industrial sponsors, associations or other private parties should be fully used and reinvested in research. The procedures for allocating and managing funds for investigators must be transparent and made less bureaucratic and therefore more rapid
(Wyatt, Faulkner-Gurstein, Cowan, & Wolfe, 2021)	Impacts of COVID-19 on clinical research in the UK: A multi-method qualitative case study	Centrally organised prioritising COVID-19 research and redeploying research staff (national decision making)	National decision making allows resources to be concentrated on studies deemed to have the greatest potential impact.
		Reduction in available research delivery staff because of redeployment to frontline care.	Shifting gears for the COVID-19 response
		Pace of work	
(Hashem, Abufaraj, Tbakhi, & Sultan, 2020)	Obstacles and considerations related to clinical trial research during the COVID-19 pandemic	Scientific and social value	*Integrity* Clinical trial design should be rigorous and analysed with full integrity. The knowledge gained should be reported completely, promptly and consistently. Research should meet all regulatory standards and conducted in an effective and safe manner.
		Resource allocation	Sound scientific research principles should not be compromised even during pandemics
		Drug repurposing	Despite the sense of urgency elicited by the pandemic, research is still subject to the same core ethical principles that govern research on human subjects.
		Evidence vs. emotional-based medicine	Institutional review bodies should be continuously informed of research progress.
		Ethics in research during the COVID-19 pandemic	To mitigate the likelihood of infection, remote monitoring in the form of telephone and video visits is strongly recommended but should be limited to essential core data and kept to a minimal frequency to avoid unnecessary burden on the investigator and trial team.
		Institutional review body efficiency	Shipments should occur in a manner that allows tracking of both transport and delivery, and participants should acknowledge receipt of shipments.
		Virtual Visits and Remote Monitoring	An alternative approach to minimising the risk of infection whilst maintaining all principles of informed consent is through virtual e-consents (information must be presented to participants in an understandable language to the participants). Study participants should also be provided with enough time to meaningfully complete the informed consent process.
		Shipments of investigational products	Alternatives to external oversight may include postponing of on-site monitoring visits, extending the period between visits and implementing video or phone visits supplemented with centralised monitoring and review.
		Hybrid models	Audits should be postponed and, when conducted, should follow social distancing roles. As the pandemic ends, robust visits and monitoring should return to the pre-pandemic processes. Priority should be given to interventions that reflect the specific needs of the patient population and are readily implementable. For patients in low-income countries, interventions should be affordable and rapidly available. During a pandemic, greater flexibility is needed for conducting clinical trials. Consider hybrid model of conducting clinical trials incorporating decentralised components only during times of crisis.
(Walker, Williams, & Bowdre, 2021)	Lessons Learned in Abruptly Switching from In-Person to Remote Data Collection in Light of the COVID-19 Pandemic	Videoconferencing	Videoconferencing: convenient, cost-effective and often user-friendly research method Facilitates real-time interactions amongst participants and researchers (building rapport) If there are challenges with emails for the completion of electronic documents alternate methods such as phone calls should be considered to collect this information.
		Internet access challenges	Limit the number of participants from to 3 or 4. This allows for substantial contribution from each participant whilst adhering to the allotted time frame.
		Incentives	Provide additional incentives for videoconference focus groups to account for the additional time and effort required to complete the demographic surveys and consent forms online, download Zoom software, answer prepared questions related to the software and log in early for troubleshooting assistance.
(Rothwell et al., 2021)	Informed consent: Old and new challenges in the context of the COVID-19 pandemic	Increased use of e-consent	Put more emphasis on the process than the document.
		Increased use of remote consent	Explore alternative mechanisms for communicating information beyond reading the text.
		Increase in barriers for obtaining signatures	Consider the use of visual images and verbal exchanges for promoting more effective informed decision-making. Provide resources for investigators to develop quality consent tools that promote understanding and address literacy concerns and training for recruiters for cultural competency and implicit bias. Consider adding to the one-time consent encounter follow-up communication. During COVID-19, re-consent may need to be obtained after capacity has been regained.
(Jayaweera et al., 2021)	Prioritising studies of COVID-19 and lessons learned	External funding sources	Consider research awards, for example, Clinical and Translational Science Awards for funding
		Funding	Funding is important for COVID-19 research.
		Infrastructure	Developing laboratory services, new diagnostics, biosafety level 2–3 laboratories and biorepositories is essential in the preparedness of a pandemic.
		Personnel	Flexible staff hiring and overtime are needed to facilitate enrolment into studies. Formation of feasibility committees to process high study proposal volume and facilitate the assessment of the feasibility and scientific merit of potential studies.
(Roshan das et al., 2021)	Challenges of developing, conducting, analysing and reporting a COVID-19 study as the COVID-19 pandemic unfolds: An online co-autoethnographic study	*Developing the study team* Deciding on the team members	Identify what further input is needed and which professional or patient groups to involve. Agree in advance on the roles and responsibilities of each team member and decide on whether or how new members will be approached or included.
		Having an advisory group	Consider having an external study advisory board with experts in the field but be clear about their roles and remit and how they will be credited in the publications.
		Patient and public Involvement (PPI)	Involve PPI early. Facilitates required training and experience. Having two people per role not only creates some differences in opinion but also ensures continuity (in case people became ill). Clear primary and deputy roles and responsibilities need to be agreed in advance of the study commencing.
		*Conducting the study* Survey platform	Use pre-existing disease-specific national registers to host new studies where possible – be aware that their pre-existing workload may delay new studies. Where registers exist, consider whether they can be adapted to include ‘control’ participants’ data also (where this is not available as part of the register). Where registers do not exist, consider developing local registries.
		ethical approval	Consider and enquire with relevant ethics committees whether amendments to previous ethical approvals will be sufficient for the new study, rather than having to apply for fresh ethical approval (which could be time-consuming).
		Ever-growing questionnaire – adding new questions	Planning for how to code inevitable changes to surveys and having all data time-stamped will enable merging and cleaning of data. Have a draft analysis plan.
		*Analysing data*	Consider in advance whether a control group is needed and how such data can be obtained.
		Ever-growing questionnaire – coding and merging data Ever-growing questionnaire – managing data analysis	Have a clear dissemination policy and a plan for how, when and how frequently to release data or report findings. Keep messages simple. Having PPI input at this stage is important.
		*Interpreting* data Control group	Register the study on an online study registry Consider having a data-sharing policy early on
		*Reporting findings or data sharing* Ongoing reporting	
(Bookman et al., 2021)	Research informatics and the COVID-19 pandemic: Challenges, innovations, lessons learned and recommendations	Telehealth (care)	Telehealth proved acceptable or even preferable for many patients and providers. It will persist after COVID-19.
		Surge in demand for virtual visits	Need for research to determine in which settings delivery of care by telehealth is equivalent, inferior, or superior compared to in-person care
		Need to rapidly develop policy, resource allocation, data sharing and secure means of patient–provider communications	Likely sustainable; not subject to any medical aid or insurance funding reversal Likely sustainable, more and better options will become available to researchers as the niche expands
		e-Consent	Research need: What are the gaps created or filled by e-Consent compared with prior practice? Which types of studies or participants are best served by e-consent
(Rania, Coppola, & Pinna, 2021)	Adapting Qualitative Methods during the COVID-19 Era: Factors to Consider for Successful Use of Online photovoice		Functional factors of an online photovoice Presence of different roles in the group Group process to make a decision Implementing empowerment Creating a favourable group atmosphere Making circular communication Factors to consider for a successful online photovoice study Employing group technological skills Presence of a climate of tension Investing greater time Technical aspects of being connected Definition of rules and strategies Developing parallel communication Absence of micro alliances Composing group
(Waterhouse et al., 2020)	Early Impact of COVID-19 on the Conduct of Oncology Clinical Trials and Long-Term Opportunities for Transformation: Findings From an American Society of Clinical Oncology Survey	A decrease in patient ability or willingness to come to the site	Keep participants informed about changes to trials and their care and remind participants to alert their research team about changes to their health
		Staff time needed to organise, implement and conduct telehealth visits	Develop formal COVID-19 standard operating procedures for clinical trials that could be repurposed with other disease outbreaks
		Limited availability of ancillary services	Leverage e-signatures for informed consent and other study documents
		Time spent in discussion with sponsors, contract research organisations (CROs), and IRBs about modifying trial procedures	Establish a system for prioritising clinical trial resource allocation (e.g. determine for which trials screening and enrolment should be maintained)
		Duplicative, inconsistent and variable communications from industry sponsors and CROs	Require remote study initiation visits and monitoring from trial sponsors and CROs Use remote safety laboratory collections, where feasible Ensure thorough documentation of changes to procedures and modifications to or deviations from protocols and use a ‘COVID-19’ tag to facilitate searching after the pandemic.

### Citation management

Importation of all citations into the web-based bibliographic manager endnote was performed. All duplicate citations were removed through the endnote functionality of identifying duplicates.

### Eligibility criteria

A two-stage screening process to assess the applicability of publications identified in the search was adopted. The first stage comprised the inclusion of publications containing keywords and phrases and those broadly describing conducting clinical research during COVID-19 to establish and describe the existing evidence base on the challenges and opportunities. The second stage involved excluding from analysis the publications that described research during COVID-19 in areas other than healthcare. However, reference lists from these publications were checked to identify additional relevant publications. As a result of lack of access to translation resources, only publications in English were included in the review.

### Title and abstract relevance screening

**FIGURE 1:**
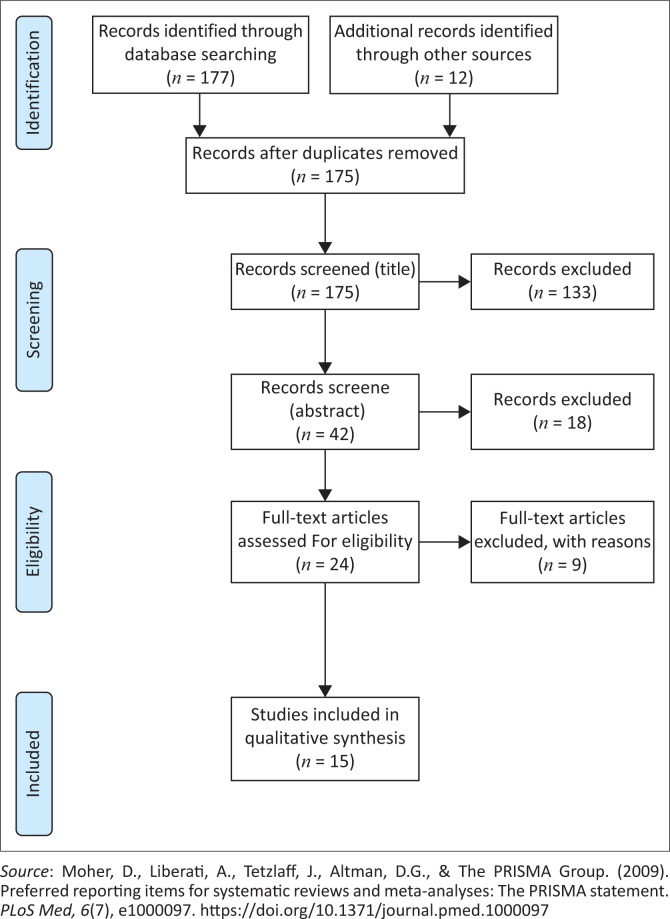
The PRISMA flow diagram describing the process of study selection.

Following Arksey and O’Malley’s (2005) efficient time management methodology, the following steps were consecutively adhered to: (1) reviewing only the titles of the manuscripts was carried out as first-level inspection; (2) reviewing of abstracts only was performed as second-level inspection and lastly (3) full manuscripts were reviewed (Figure 1). The researchers utilised the research team’s previously developed and pretested abstract relevance screening spreadsheet, which had a reviewer agreement (overall kappa) greater than 0.8 – representative of a high level of agreement (Viera & Garrett, 2005). The titles, abstracts and full manuscripts were independently screened by all three researchers, with the process developed to facilitate triangulation during the process of data selection and analysis. Where research titles did not have abstracts, these were all included in the full article review stage of the data characterisation phase. Regular online communication between the researchers was maintained during the entire process to make sure that conflicts were resolved, with one author (BS) making the final decision when disagreements were found. A high level of agreement was found with the overall kappa of 0.81. Post the data analysis, two independent reviewers, a PhD fellow and a postdoc fellow were recruited to review the manuscript with its accompanying supportive data to validate the findings reported in the scoping review.

### Data characterisation

Following the completion of the title and abstract inspection stage, all relevant citations for this scoping review on the conduction of clinical research during COVID-19 were acquired for later full articles review. The researchers developed a spreadsheet where the relevance of each publication was confirmed and where details of the article such as author and publication year, article title, context, challenges, recommendations or opportunities and conclusions were documented. The characteristics of each publication were extracted by all three researchers. Publications that did not meet the minimum eligibility criteria were then excluded at this phase. Open engagement between the researchers, in line with Levac et al.’s (2010) framework, for internal consistency and for resolution of prevailing conflicts between them occurred. Furthermore, the researchers also ensured that the articles included were consistent with the stated research question and purpose following their independent reviews.

### Data summary and synthesis

Data were recorded in a spreadsheet and imported into Microsoft Excel 2016 (Microsoft Corporation, Redmond, WA, USA) for descriptive narrative analysis.

Initially, a total of 189 articles were identified for potential analysis in this study. During the collation and organisation of the studies part of the scoping review, 14 studies were deleted as they were duplicates; consequently, only 175 studies were then considered. Of the 175 studies that remained, 151 were eliminated based on the titles and abstracts that were considered to not be in line with the focus of this study. Subsequently, 24 studies were evaluated for eligibility and from these 9 were omitted as they failed to meet the inclusion criteria of this study. Ultimately, 15 articles were included for analysis in this study (see Figure 1).

### Ethical considerations

This scoping review followed all ethical standards for a study that does not involve direct contact with human or animal participants, including reflexivity and informed subjectivity, audience-appropriate transparency and purposefully informed selective inclusivity (Suri, 2020). Due to the nature of this study being based on published articles (secondary data), there was no need to seek ethical clearance.

## Results and discussion

As depicted in Table 1, 15 publications were included in this review following them to meet the inclusion criteria revealed significant challenges with conducting research in the COVID-19 era, with important lessons learned and numerous opportunities that have relevance for this pandemic era and beyond. These publications were diverse because they included opinion pieces and commentaries, research reviews, as well as various types of empirical studies, for example, single group prospective, cross-sectional, clinical trials, etc., all engaging the research question. Findings are presented and discussed under 10 major themes that were identified: (1) the importance of having processes in place to balance research priority, speed and high quality; (2) approval processes can be efficient without compromising the research integrity process; (3) need for flexibility in research protocols and designs currently and beyond COVID-19; (4) need to interrogate participant recruitment, participant participation and informed consent within the realm of Information and Communication Technology (ICT) currently and beyond COVID-19; (5) Remote working practices for data collection unavoidable but highly recommended; (6) intensified efficient use of ICT for research processes; (7) research informatics and ICT use and innovation (telehealth) has its problems; (8) challenges with actual interventions; (9) challenges with data capturing, analysis and storage and (10) challenges with research findings sharing or publishing.

Findings under these 10 themes suggest an emergence of new approaches to conducting research since the start of COVID-19 pandemic that the South African Speech-Language Pathology and Audiology professions can learn from. The significance of developing a sustained research infrastructure and research workforce that can continue research under PHE contexts with efficient training and funding for researchers who are integrated into the healthcare workforce across the scopes of practice was highlighted.

### Importance of having processes in place to balance research priority, speed and high quality

During the COVID-19 pandemic, obvious and inevitable prioritisation of all efforts towards curbing the spread and managing COVID-19 was witnessed globally and this included prioritisation of research on this virus (Jayaweera et al., 2021; Wyatt et al., 2021). Despite the prioritisation of COVID-19 and research on it, Weiner et al. (2020) highlighted that during PHEs, such as the COVID-19 pandemic, several measures need to be in place to ensure that time and cost efficiency challenges are prevented and mitigated. These measures include (1) consideration of crisis standards for research along with the ongoing PHE, with accompanying appropriate PHE training for researchers; (2) consideration of dedicated funded core workforce of PHE multidisciplinary researchers in the research site organogram, as well as funded research infrastructure, to plan, consult, review, monitor, interpret studies, guide appropriate clinical use of data and inform decisions regarding effective use of resources for PHE research – in the midst of other ongoing research that is not COVID-19 related; measures should be taken to include sufficiently prepared and remunerated research support professionals in training and funding plans to enhance the management of clinical trials and support the date collection processes (e.g. data manager or study coordinator); (3) consideration of research funding and appropriate infrastructure, with concreted efforts towards securing external private party funding, where transparent and less bureaucratic procedures for allocating and managing funds for investigators are well documented; (4) consideration around processes to be adopted where reduction in available research delivery staff because of redeployment to frontline care has to be carried out, with centrally organised prioritising COVID-19 research and redeploying research staff recommended and (5) collaboration amongst national policymakers, the pharmaceutical industry, opinion leaders, patient advocacy groups and regulatory agencies for appropriate resource allocation. All these measures, along with the rest of the recommendations put forward in this article, would ensure that there is an efficient balancing of research priority, speed and high quality.

Roshan das et al. (2021) recommended that in developing the study team, it is important to (1) decide on the constituency of the team members – and that this should be multidisciplinary in nature as this makes the team stronger, but small enough to facilitate quick and efficient resolution of conflicts. Therefore, these authors suggest that for efficient but diverse functioning and for succession planning, two people be appointed per role; (2) constitute an external study advisory board consisting of field experts, with clearly defined roles and responsibilities, as well as agreed-upon manner of accreditation in future publications emanating from the research group; (3) have a patient and public involvement (PPI) that is argued to ensure that researchers keep the needs of the patient at the forefront – and it is advised that PPI must occur early in the study as this facilitates the acquisition of the requisite training and experience and furthermore, PPI can aid with publicity for the study thus improving recruitment and (4) have written agreements drawn up about the roles and responsibilities of each team member.

### Approval processes can be efficient without compromising the research integrity process

With all the challenges to research created by COVID-19, Weiner et al. (2020) and Wyatt et al. (2021) stressed the importance of having a well-established coordinated review and study process to make the best use of constraint resources. Hashem et al. (2020) cautioned that regardless of the sense of urgency caused by the pandemic, the same core ethical principles that govern research on human subjects are still expected to be adhered to. The current scoping review revealed that the COVID-19 era exposed that for such review and study activation processes, including ethics or research approvals and institutional review bodies (IRBs), it is feasible to considerably reduce the administrative, regulatory and time costs that are involved in coordinating, registering and conducting research, specifically trials (Bailey et al., 2020; Cagnazzo et al., 2021; Hashem et al., 2020). Evidence revealed that simplified approval processes are possible and these streamlined approval procedures that were devised for COVID-19 trials can be continued beyond this pandemic era. Cagnazzo et al. (2021) argued that these procedures can be employed in different types of clinical research, over and above clinical trials.

Approval processes can be efficient without compromising the research integrity process through IRBs: (1) ensuring that the standard of ethical review is not relaxed; (2) ensuring that informed consent is always secured, despite the PHE, whilst ensuring efficient use of resources; (3) considering issues such as strict exclusion and inclusion criteria, participant compensation and reimbursements, as well as well-defined risks of the study to vulnerable participants; (4) improving their expediency during PHEs and (5) strengthening online processes such as placing template case report forms and ethics forms online for online entry and modification and being continuously informed of research progress (Bailey et al., 2020; Cagnazzo et al., 2021; Hashem et al., 2020; Rania et al., 2021; Roshan das et al., 2021; Shamsuddin et al., 2021; Walker et al., 2021; Waterhouse et al., 2020).

### Current and beyond COVID-19 need for flexibility in research protocols and designs

As a result of the unpredictable nature of COVID-19, with its numerous waves and ever-developing variants (Lone & Ahmad, 2020; The World Economic Forum, 2020) influencing research processes and timelines as well as intervention plans, research plans deviations are inevitable. This implies the need for flexibility in research protocols and designs as recommended by Bailey et al. (2020). Fleming et al. (2020) believed that protocol deviations may lead to increased flexibility in the design of new trials and Hashem et al. (2020) further stated that during a pandemic, greater flexibility is needed for conducting clinical trials. These authors stress that, within this flexibility, research teams must have ‘appropriate study designs, collaboration, patient registries, automated data collection, artificial intelligence, data sharing and ongoing consideration of appropriate regulatory approval processes’ (Weiner et al., 2020, pp. 148–149). They further recommended that researchers must consider hybrid models of conducting research, models that incorporate decentralised components (e.g. remote work practices, ICT use, home visits, etc.) only during times of crisis, such as during a PHE like COVID-19.

It is important to plan for possible changes to the research methods and design. For example, Roshan das et al. (2021) gave an example of the manner in which a possibility of questionnaires requiring changing, researchers must be prepared for this change and contemplate on what measures or processes must be put in place to enable or facilitate the change at the appropriate time. Another example these authors provide is planning ahead on methods that will be employed to code inevitable changes to study tools, such as surveys, as well as ensuring that all data are time-stamped, which will facilitate data cleaning and merging.

### Current and beyond COVID-19 need to investigate participant recruitment, participation and informed consent within the realm of information and communication technology

Numerous publications reviewed (Bailey et al., 2020; Cagnazzo et al., 2021; Fleming et al., 2020; Hashem et al., 2020; Roshan das et al., 2021; Rothwell et al., 2021; Shamsuddin et al., 2021; Waterhouse et al., 2020) highlighted significant gaps in standard processes involved in patient recruitment, participant participation and informed consent, under pre-COVID-19 conditions, with these intensified within the ICT realm created by remote working. These gaps that straddle between remote working practices and intensified use of ICT for research processes raised an important theme that of a need to carefully scrutinise these research processes for current and future research. Jayaweera et al. (2021) and Cagnazzo et al. (2021) argued that constant review of evolving knowledge, in this case knowledge that emerged because of the COVID-19 pandemic, must be utilised to refine and enhance research processes; thus, ensuring that the future of research centres on preparedness and on not repeating past or current errors.

Cagnazzo et al. (2021) highlighted that patient recruitment and participation require re-consideration to alternative measures that have the potential to enhance patient participation in research. Four key considerations that these authors put forward that can serve as alternatives approaches are: (1) offering reimbursement of expenses incurred by participants and their families and caregiverss, such as travel, examinations, procedures, without limitation to rare disease clinical trials only; (2) facilitating remote participants visits (e.g. video, telemedicine, phone); (3) provide incentives for the possibility to conduct procedures at the participant’s home – home visits (e.g. drug administration, blood sample taking, administration of questionnaires and function tests, cochleovestibular monitoring, etc) whilst safeguarding the participant’s anonymity and (4) allow the use of healthcare facilities (e.g. laboratory for blood analyses) other than the reference centre, with strict study protocol monitoring.

### Remote working practices for data collection unavoidable but highly recommended

As part of non-pharmaceutical interventions to manage the spread of COVID-19 (Imai et al., 2020; Perra, 2021), including the in-person interactions restrictions created significant challenges with research (Bookman et al., 2021; Fleming et al., 2020; Walker et al., 2021). These challenges necessitated the need for remote working practices for research, practices that can be carried forward to beyond the COVID-19 era. Remote working practices were adopted to facilitate research planning, research review and approval (IRB communications), participant recruitment, patient to participant contact, interventions and data collection, site visits and training (Bailey et al., 2020; Cagnazzo et al., 2021; Hashem et al., 2020). For example, Cagnazzo et al. (2021) encouraged the facilitation of remote patient visits, such as video conferencing, telehealth, use of telephones, etc. Hashem et al. (2020) highlighted that to mitigate the likelihood of infection, although remote research processes, such as monitoring, through the use of ICT in the form of video visits and telephone calls are crucial, it is important that this practice should be restricted to essential core data and maintained to an absolute minimal frequency in order to evade redundant workload on the research team. Walker et al. (2021) argued that videoconferencing is a convenient, cost-effective and often user-friendly research method and recommend the use of online photovoice – as this facilitates real-time interactions amongst participants and researchers (building rapport). Current reviewed studies highlighted the importance of identifying needs that can be addressed before conducting remote research, as well as establishing whether this form of data collection is suitable for the research question and population of interest (Roshan das et al., 2021; Walker et al., 2021). This includes the facilitation of remote monitoring of the research and verification of source data (Cagnazzo et al., 2021; Hashem et al., 2020).

### Intensified efficient use of information and communication technology for research processes

As a result of the enforced remote working practices, the use of ICT for research processes had to be intensified during COVID-19, however, these practices can be taken forward beyond this pandemic era (Bailey et al., 2020; Bookman et al., 2021; Hashem et al., 2020; Roshan das et al., 2021; Rania et al., 2021; Shamsuddin et al., 2021; Walker et al., 2021). If adapted suitably, ICT has been demonstrated and recommended to possess the potential to transform the entire research process, including increasing awareness of and access to clinical studies (Bailey et al., 2020; Shamsuddin et al., 2021; Rothwell et al., 2021). Through the use of remote interfaces and apps as well as telehealth technologies, be it synchronous, asynchronous or hybrid models (Khoza-Shangase et al., 2021; Sebothoma et al., 2021), there have been significant research enhancing changes such as participant recruitment, communication with patients regarding eligibility for various studies and for informed consent and for monitoring symptoms and drug side effects for clinical trials (e.g. drug safety trials) and longitudinal studies (Bailey et al., 2020; Roshan das et al., 2021; Rothwell et al., 2021). For example, COVID-19 has raised awareness amongst key stakeholders involved in research about the limits of informed consent and offers the research community a rare opportunity to advance significant change that can meaningfully enhance informed decision-making for research and research access (Hashem et al., 2020; Rothwell et al., 2021; Shamsuddin et al., 2021; Waterhouse et al., 2020).

As far as informed consent is concerned, Hashem et al. (2020) and Roshan das et al. (2021) stressed that during pandemics such as the COVID-19, researchers must take into cognisance the strong risk of infection transmission through paperwork used in information sheets, consent forms, questionnaires etc. This risk can be mitigated through the application of data acquisition, capture and storage processes that are performed electronically, thus raising the value and challenges of electronic acquisition of informed consent.

### Research informatics and information and communication technology use and innovation (telehealth) has its problems

This review revealed numerous opportunities and challenges with informed consent that are linked to ICT (Bailey et al., 2020; Walker et al., 2021). Depending on how these are addressed, they can be challenges or opportunities: (1) increased use of e-consent and increased use of remote consent, (2) increase in participants who do not speak English – a language that is often used in research, (3) increase in challenges with obtaining signatures and increased use of waiver of signatures, (4) increased use of legally authorised representatives, (5) increased use of clinician team to consent and (6) increase in re-consenting when either participant capacity has returned following intervention interruption because of COVID-19 or research disruption because of lockdowns, etc. (Bailey et al., 2020; Bookman et al., 2021; Rothwell et al., 2021; Waterhouse et al., 2020).

The current researchers, as also recommended by Rothwell et al. (2021), argue that during COVID-19 and beyond, the practice of informed consent acquisition should place more emphasis on the process than the document itself. These authors suggest that this entails the exploration of alternative means for communicating information outside reading the text. Alternative strategies include the use of verbal exchanges and visual images that foster more effective informed decision-making (Rothwell et al., 2021). Thus, it is important that resources should be made available to research teams to develop quality consent tools and methods that advance proper understanding and address literacy and linguistic concerns (Khoza-Shangase & Mophosho, 2018, 2021; Rothwell et al., 2021; Hashem et al., 2020). Rothwell et al. (2021) suggested that institutions provide resources that facilitate consent translation and context-relevant interpreters. Resource allocation for this aspect should also take into consideration training of recruiters and the relevant research team members on cultural competency and implicit bias (Roshan das et al., 2021; Rothwell et al., 2021). Roshan das et al. (2021) also recommended the use of PPI members in this role and argued that they can help with the development of appropriate research tools as they are intimate with the public and patient needs. Lack of these resources significantly hinders the research process and may cause harm by creating a barrier to inclusive access to research and therapeutic interventions (Rothwell et al., 2021).

Placing more emphasis on the informed consent process rather than the document itself also requires that interruptions brought about by COVID-19 be considered during the development of the consent protocol. The fact that re-consent could be required following study interruptions, disruptions or stoppages – after capacity has been regained, Rothwell et al. (2021) recommended that researchers should consider adding follow-up communication to the one-time consent encounter.

Further challenges with ICT involve challenges with research informatics, ICT use and innovation (e.g. telehealth, research virtual visits). Key to these challenges, over and above-informed consent, is the well-documented bias of this model of research to those with internet access, thus excluding a significant part of individuals, particularly in LMICs (Shamsuddin et al., 2021; Walker et al., 2021). Linked to, but not exclusive to this access challenge, is familiarity with the internet and the e-world, including skills such as ability to log in to check e-mails for research deadlines and logging in to participate in videoconferencing or interviews, etc. For the researchers and all participants, familiarity with the internet has an influence in issues such as the number of participants who can be included in an e-interaction or visit, this affects the time allocation for each event because it has an impact on the time allotted for the activity (Hashem et al., 2020; Rania et al., 2021; Walker et al., 2021; Weiner et al., 2020). Another important challenge with research informatics involves online data handling storage and destruction (Bookman et al., 2021; Hashem et al., 2020; Shamsuddin et al., 2021); specifically (1) the ethical implications for recording online and this includes a private recording of the event by participants – particularly when it involves more than one participant as is the case with focus groups; (2) storage of recordings and destruction of data, as well as (3) challenges with the establishment of rapport amongst participants and between participants and researchers (Hashem et al., 2020; Shamsuddin et al., 2021; Walker et al., 2021).

### Challenges with actual interventions

Challenges with actual interventions because of factors such as potential delays or pause in enrolment of participants, challenges with monitoring adherence increase the risk of bias; later re-initiation of enrolment and its implications for the research protocol; interruption of intervention delivery and data collection, analytical issues in protecting research integrity, etc. (Bailey et al., 2020; Fleming et al., 2020; Hashem et al., 2020; Rothwell et al., 2021). Findings from the review raise a need for home visits for intervention delivery and monitoring whilst in full personal protective equipment, as well as efficient maintenance of contact with research participants by the research team for participant retention for later re-initiation (Bailey et al., 2020; Cagnazzo et al., 2021; Fleming et al., 2020). Furthermore, Cagnazzo et al., (2021) and Hashem et al. (2020) suggested that the feasibility of interventions should be carefully evaluated before studies are conducted. These authors posit that priority should be given to interventions that reflect the explicit needs of the patient population where the studies are being conducted and interventions that are easily implementable. In LMICs, current authors recommend that interventions should be rapidly available and affordable.

### Challenges with data capturing, analysis and storage

As a result of the challenges with data collection caused by interruptions, pauses, re-entering and adherence to monitoring because of COVID-19, data capturing may reveal incomplete or missing data, and the consequent need for revision of the statistical methods planned are inevitable (Fleming et al., 2020; Roshan das et al., 2021). Roshan das et al. (2021) and Waterhouse et al. (2020) recommended that modifications to data collection and revisions to planned statistical procedures should be discussed with all relevant stakeholders including clinicians, operational staff, data management teams and statisticians and that these changes should be well documented and reviewed by appropriate authorities and committee, for example, IRBs (Bailey et al., 2020; Hashem et al., 2020; Waterhouse et al., 2020).

As far as data storage is concerned, Shamsuddin et al. (2021) raised critical considerations around the ethical implications for recording online, such as recording of sessions, as well as informed consent for recordings. These authors offer guidance on how to address these challenges. Firstly, for addressing online recording, they suggest strict adherence to the ethical principles of justice, beneficence and respect for persons, whilst ensuring that all necessary precautions are in place for data collection, where participant confidentiality and privacy are respected and protected. Furthermore, these authors suggest that suitable online recording tools should be utilised and the intention to record online be clearly communicated to the IRB. Secondly, to ensure informed consent for recordings, Shamsuddin et al. (2021) suggested that researchers should make it clear during the process of obtaining consent that recordings cannot be removed after participation, thus limiting the chance of different consent for recording. In addition, a clear confidentiality statement must form part of the participant information sheets and consent forms and these information sheets should also disallow participant recording of the research session using their own recording devices.

As far as storage and destruction of recordings is concerned, Shamsuddin et al. (2021) offered numerous recommendations. Firstly, researchers should make an informed choice about what type of software to use for storage, for example, whether to use the web conferencing system’s built-in function or to use software external to the web conferencing system used during data collection. Secondly, researchers must be knowledgeable about the recording facility’s privacy policy, for example, some hosting platforms such as Blackboard Collaborate™ Ultra and Zoom™ store recordings on their platform (i.e. cloud storage), and such recordings can later be downloaded to the researcher’s computer for secure long-term storage. Thirdly, researchers must strengthen their study data security for recordings stored on the host provider’s platform by having password protection that ensures that they have complete control over who has access to it. Finally, researchers must decide on how long recordings should be stored and must also ensure that the methods for data destruction are appropriate. Most importantly, they must make sure that data stored on these virtual host platforms are destroyed, so that this data does not get used for market research purposes.

### Challenges with research findings sharing or publishing

As a result of the emergency created by COVID-19, sharing and publishing of research findings became critical as global evidence on prevalence or incidence, symptomatology, interventions and treatment outcomes was required. Park et al. (2021) and Bailey et al. (2020) emphasised the obvious advantages of preprint servers and the merits of a faster peer review process, resulting in quicker dissemination of findings that can be utilised to inform policies and fast track the research and development (R&D) process for COVID-19 interventions and vaccines. This review indicates that research findings sharing or publishing can be faster, with most journals offering preprints. However, an identified challenge with this process was compromised poor quality of studies with many non-peer-reviewed preprints with reduced standards (Bailey et al., 2020; Park et al., 2021). Park et al. (2021) and Bailey et al. (2020) lamented that the differentiation between peer-reviewed publications and preprints with apposite oversight became distorted. This practise, these authors believe, has a significant impact on the scientific community and the public. Thus, during and beyond COVID-19, whilst facilitating fast speed of dissemination, processes should be in place to ensure that standards are maintained. Bailey et al. (2020) stated that such a safeguarding process entails: (1) transparency in data sources and analysis procedures, (2) repeatability and reproducibility and (3) vigorous peer-review process.

As far as the transparency is concerned, it is crucial that researchers inform journals of any changes to research protocols and statistical procedures that were because of COVID-19-related impacts such as study commencement delays, interruptions, pauses, late re-enrolments and so on. Fleming et al. (2020) suggested that these COVID-19-induced modifications and so on must be described in detail in the methodology section of the publication, with any protocol amendments highlighted in the cover letter submitted at the time of the article submission to the journal (Bailey et al., 2020).

As a result of the changing nature of the COVID-19 pandemic, the reviewed studies recommend that findings or data sharing and reporting should be ongoing, with very clear and simple messages. Roshan das et al. (2021) suggested the importance of incorporating PPI input at this stage to ensure simplicity and clarity. This, it is highlighted, can only occur if research units have clear dissemination policies and plans for how, when and how frequently data or report findings will be released. Furthermore, this process will be safeguarded by ensuring the protection of intellectual property and having clear copyright statements that provide contact details of key authors to respond to data sharing requests, with clear policies around authorship. Roshan das et al. (2021) and Bookman et al. (2021) recommended that data sharing and authorship policies should be in place at the commencement of research projects, whilst Roshan das et al. (2021) recommended that researchers register their studies on an online study registry, such as ClinicalTrials.gov, to protect intellectual property. Furthermore, these authors recommend that pre-existing disease-specific national registers be utilised to host new studies where possible, but caution that researchers must remain awake to the possibility that their pre-existing workload may lead to delays to new studies. Where pre-existing registers do not exist, these authors suggest developing local registries. Furthermore, they recommend that where pre-existing registers exist, researchers should deliberate and query with relevant ethics committees if simply submitting amendments to earlier ethical approvals would be adequate for their new study, instead of re-applying for new ethical approval, thus, saving time. Alternatively, emergency, fast-track ethical approval processes should be explored from universities and relevant institutions. In addition, Roshan das et al. (2021) suggested that in such scenarios, researchers should also establish links with other researchers working in related studies to agree on common minimum standards or contents of research tools, such as questionnaires – which may be of benefit to everyone involved.

## Conclusion

The current scoping review aimed at answering the question ‘what has been published about conducting clinical research during the COVID-19 pandemic?’ Findings have revealed both challenges and significant opportunities spanning from the inception of research teams, setting up research protocols, obtaining institutional and ethical approval, all the way to actual interventions, data collection and analysis, data recording and storage, sharing research findings and publishing. These findings are all whilst considering remote working conditions as imposed by COVID-19, with the use of ICT for research revealed to have intensified. The findings of this study are presented under 10 themes that emerged from the data. The current findings not only highlight important considerations for research during a pandemic but also beyond, where ICT and telehealth can play a significant role in increasing access to both research participants recruitment and participation and provision of interventions remotely, be it for research purposes or for clinical care – particularly in LMICs where challenges with the healthcare workforce are well documented. Lessons about access in research are important to take forward as they might facilitate access to larger and more diverse participants for clinical studies, affording researchers data that may be more relevant and findings that are more generalisable. These considerations are critical for the Speech-Language Pathology and Audiology professions to deliberate on for current and future (beyond COVID-19) clinical research planning. Regardless of the fact that some studies included in this review are from a purely medical perspective, the challenges and opportunities identified in those studies are similar to and are transferable to the field of Speech-Language Pathology and Audiology.
